# BFR2: a curated ribosomal reference dataset for benthic foraminifera

**DOI:** 10.1038/s41597-024-04137-8

**Published:** 2024-11-27

**Authors:** Maria Holzmann, Ngoc-Loi Nguyen, Inès Barrenechea Angeles, Jan Pawlowski

**Affiliations:** 1 Department of Genetics and Evolution, University of Geneva, 1211 Geneva 4, Switzerland; 2 Department of Paleoceanography, Institute of Oceanology Polish Academy of Sciences, 81-712 Sopot, Poland; 3 Department of Geosciences, UiT the Artic University of Norway, 9010 Tromsø, Norway

**Keywords:** Evolution, Ecology, Taxonomy

## Abstract

Benthic foraminifera are one of the major groups of marine protists that also occur in freshwater and terrestrial habitats. They are widely used to monitor current and past environmental conditions. Over the last three decades, thousands of DNA sequences have been obtained from benthic foraminiferal isolates. The results of this long-term effort are compiled here in the form of the first curated benthic foraminiferal ribosomal reference dataset (BFR2). The present dataset contains over 5000 sequences of a fragment of the 18S rDNA gene, which is recognized as the DNA barcode of foraminifera. The sequences represent 279 species and 204 genera belonging to 91 families. Thirteen percent of these sequences have not been assigned to any morphologically described group and may represent species new to science. Furthermore, forty-five percent of the sequences have not been previously published. The BFR^2^ dataset aims to collect all DNA barcodes of benthic foraminifera and to provide a much-needed reference dataset for the rapidly developing field of molecular foraminiferal studies.

## Background & Summary

Foraminifera are one of the most diversified group of protists (microbial eukaryotes). They are characterized by the presence of a specific type of pseudopodia (granuloreticulopodia), and a test (shell), which can be calcareous, agglutinated, or organic. They are widely distributed in marine and freshwater environments. The group counts 8,896 modern and 39,976 fossil species^1^ (www.marinespecies.org). The majority are benthic species that live epifaunal or infaunal. About 50 modern species are planktonic. Foraminifera represent the most important group of microfossils, widely used in paleostratigraphic and paleoclimatic studies^2,3^. The modern foraminifera are also widely used in biomonitoring, as bioindicators of anthropogenic activities^4,5^. They have been shown to be highly sensitive to environmental changes caused by natural and anthropogenic factors, such as climate change, anoxia, organic enrichment, or pollutants^6–10^.

Traditionally, foraminifera are identified by the morphological features of their test. Foraminiferal morpho-taxonomy is largely based on the composition and structure of the wall and the form and ornamentation of the test^11,12^. The advent of molecular systematics has fundamentally changed our knowledge of foraminiferal diversity, revealing the importance of soft-walled, single-chambered monothalamous foraminifera that had been largely overlooked by microfossil-oriented foraminiferal research^13,14^. Molecular studies have also expanded the range of habitats, in which foraminifera occur, showing that they live not only in marine habitats but also in freshwater and soil environments^15^. At the species level, molecular studies have demonstrated high levels of cryptic diversity in virtually all foraminiferal groups, showing that most morphospecies are composed of several cryptic species that can only be identified based on DNA sequences^16^. Microscopic studies and single cell sequencing also show that foraminiferal tests can be colonized by alien foraminiferal species, known as squatters which further complicates the correct identification of obtained sequences^17,18^.

To assess the cryptic diversity and to aid in the identification of foraminiferal species, DNA barcodes specific to foraminifera have been developed^19^. A fragment of the 18S rDNA gene was chosen as the foraminiferal barcode. The fragment is composed of six hypervariable regions, three of which are specific to foraminifera, allowing the discrimination of closely related species or populations^20^. Although high levels of intragenomic polymorphism have been reported in some species, this does not seem to affect its use for species identification^21^. The mitochondrial COI gene recently proposed as an alternative foraminiferal barcode appears to be less resolutive than the 18S gene^22^.

The 18S DNA barcodes have been successfully used to revise the morphology-based taxonomy of benthic foraminiferal species. The diversity of several genera (e.g. *Ammonia*) has been greatly expanded^23,24^. Numerous new species have been described, based on phylogenetic analyses of the 18S gene^25,26^. A short fragment of the barcoding gene has also been used in metabarcoding to assess the environmental diversity of foraminifera^27^. Metabarcoding studies revealed a large number of unknown foraminiferal species, most of which belong to soft-walled or naked monothalamid taxa^28^. The majority of these taxa could not be assigned to any reference sequence^29^. The lack of a unified 18S reference library for benthic foraminifera seriously hampers the identification of foraminiferal environmental sequences.

The present paper allows to overcome this limitation by providing an open-access, curated dataset for benthic foraminifera. The dataset is a continuation of the efforts to establish DNA barcode reference libraries for different groups of protists^30^. These efforts have been initiated by the development of the Protist Ribosomal Reference dataset (PR^2^) by Guillou, *et al*.^31^. DNA barcoding reference libraries also exist for diatoms^32^, ciliates^33^, and dinoflagellates^34^. Among foraminifera, only planktonic species ribosomal reference sequences have been catalogued^35,36^. Here, we present the first DNA barcode library of benthic foraminifera.

**Fig. 1:**
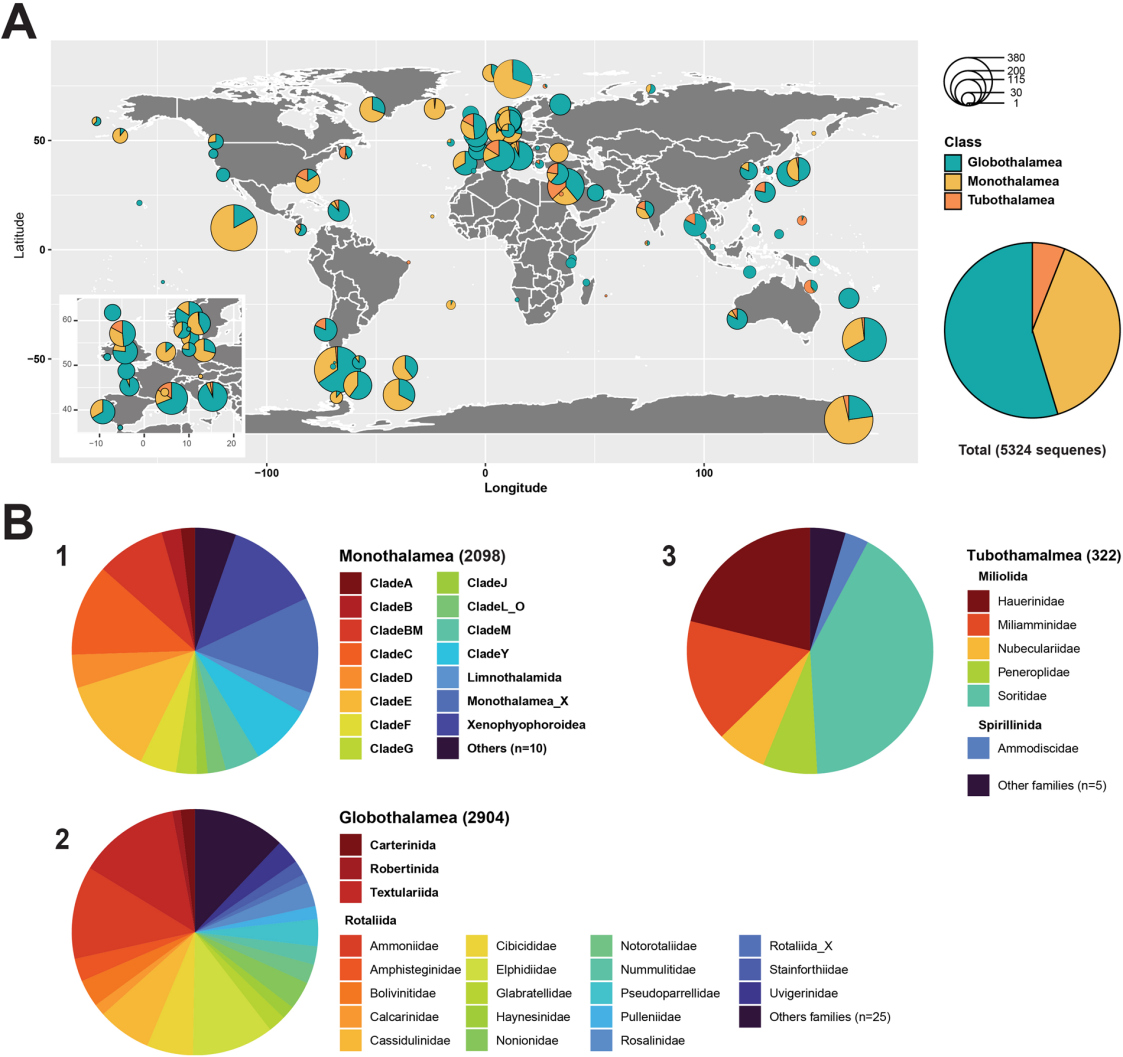
Distribution of foraminiferal 18S rDNA barcodes by region. The inset map focuses on the European region with its many sampling sites (**A**). Piecharts indicate the taxonomic composition of the three main classes Globothalamea, Tubothalamea, and Monothalamea (**B**). Classes are divided into families or clades (1, 2, 3) and one order (1).

The dataset includes 5324 sequences of the 18S rDNA gene. The sequences were obtained from foraminiferal specimens collected all over the world (Fig. 1A). Sequences from high latitude regions (Arctic, Antarctic) and deep-sea settings are particularly well represented in our dataset which is due to a sampling bias. The taxonomic composition of the dataset comprises three major classes: Globothalamea, Tubothalamea, and Monothalamea, represented by 2904, 322, and 2098 sequences respectively (Fig. 1B). The fourth major class, Nodosariata, is represented by a single sequence only. Within Globothalamea, Rotaliida are the most important group (2428 sequences), with Ammononiidae (356 sequences) and Elphidiidae (313 sequences) being the most abundant rotaliid families. Within Tubothalamea, most sequences (83) were obtained for the genus *Sorites*. Monothalamean groups particularly well represented are Clade C (253 sequences) and Xenophyophoroidea (264 sequences). The class Monothalamea also comprises numerous sequences (349) that cannot be assigned to any formally described taxon.

## Methods

### Material collection

Most of the sequences (4457 of 5324) were obtained from foraminiferal specimens (isolates) collected by Jan Pawlowski and collaborators over the last 30 years. The collection contains more than 22.000 DNA extracts from individual specimens stored in the foraminiferal DNA collection of the Department of Genetics and Evolution at the University of Geneva (curated by Maria Holzmann and Jan Pawlowski). Most DNA extracts are from marine specimens sorted from sieved sediments in seawater. Subtidal, bathyal, abyssal and hadal samples originated from box corers and multicores or epibenthic sledges. After collection, subsamples of the oxygenated sediment top layer were removed using spoons and sieved on screens with various mesh sizes, 350 μm, 300 μm, 250 μm, 125 μm and 63 μm, using cooled sea water^18,25,26^. At intertidal locations, oxygenated surface sediment samples were obtained using spoons and containers with sediment samples from each site were filled with natural sea water^23^. For all samples, the residues in seawater were transferred into Petri dishes and Foraminifera that appeared alive (generally based on the presence of cytoplasm) picked out using a pipette or fine brush. Foraminiferal specimens were identified morphologically using a Stereomicroscope equipped with a camera prior to extraction and taxonomically assigned. Most of the non-marine foraminifera were obtained from freshwater surface sediment samples and water plants and could be maintained for some time in laboratory cultures fed with algae and baker’s yeast^37^. Organic-walled or agglutinated specimens were preserved in RNAlater or guanidine; hard-shelled specimens were dried at ambient temperature^38^. Specimens were routinely photographed before extraction.

### DNA extraction, PCR amplification and sequencing

**Fig. 2:**
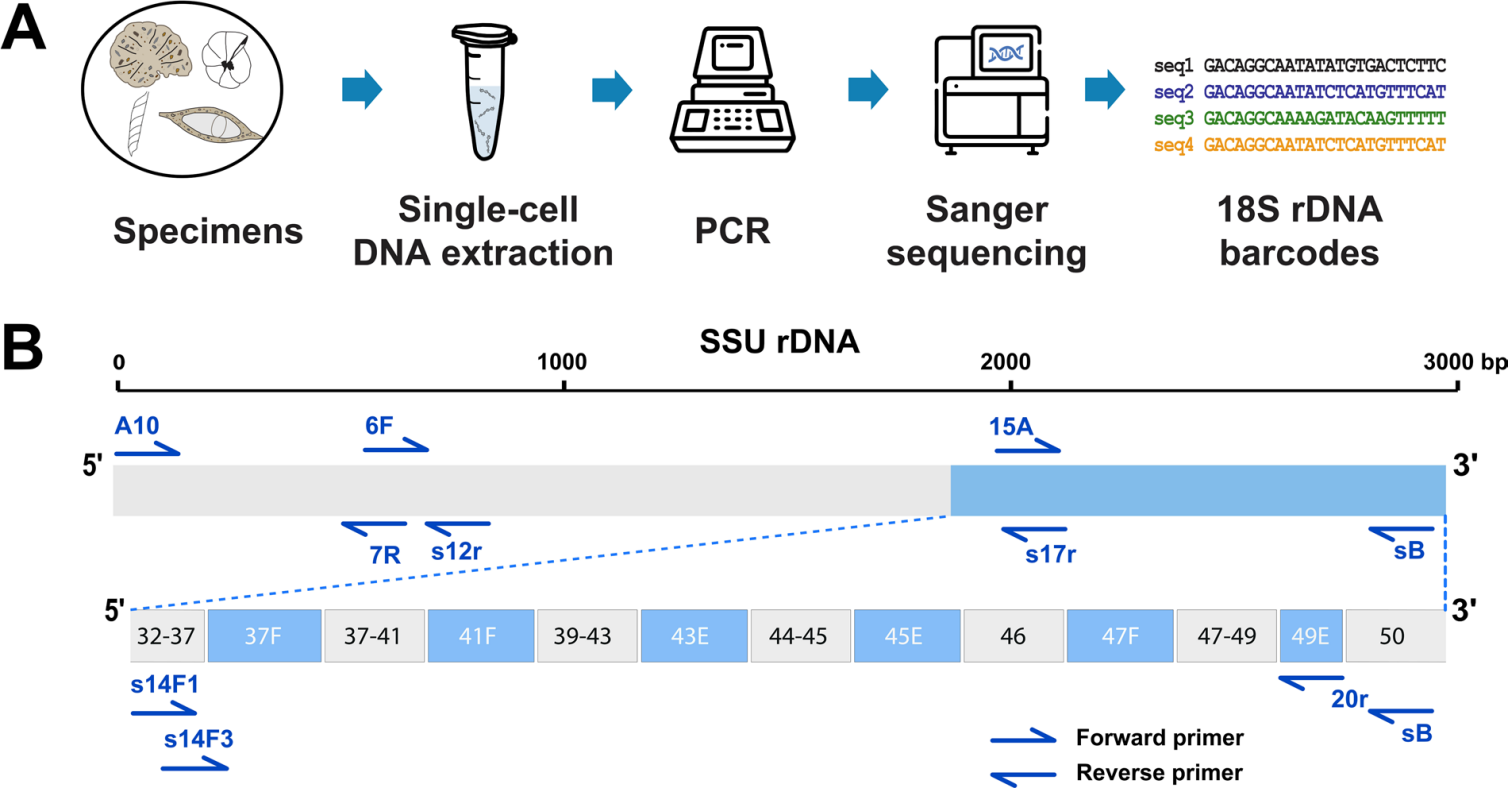
Workflow of DNA barcoding technique (**A**) from specimen collection to obtaining benthic foraminiferal barcodes, and scheme of the 18S rDNA barcoding fragment in correlation with the position of amplification primers (**B**).

DNA was extracted from single specimens using either guanidine lysis buffer^39^ or DNeasy Plant Mini Kit according to the manufacturer’s instructions. Semi-nested PCR amplification was carried out for all isolates^19^. The standard barcoding fragment is obtained using primers s14F3 (5′ ACG CAM GTG TGA AAC TTG 3′) and sB (5′ TGA TCC TTC TGC AGG TTC ACC TAC 3′) for the first and primers s14F1 (5′ AAG GGC ACC ACA AGA ACG C 3′) and sB for the second amplification. In some cases, when the PCR did not yield positive results, the reverse primer sB was replaced by primer s20r (5′ GAC GGG CGG TGT GTA CAA 3′) or s17r (5′ CGG TCA CGT TCG TTG C 3′) (Fig. 2). In addition, complete 18S sequences were obtained for 131 isolates (104 Globothalamea, 18 Monothalamea, 8 Tubothalamea, 1 Nodosariata). Complete 18S sequences of Tubothalamea are more than 2000bp long, for Globothalamea and Monothalamea these sequences are more than 3000 bp long. The complete SSU rDNA gene was amplified in three overlapping fragments. For the 5′ end fragment primers A10 (5′ CTC AAA GAT TAA GCC ATG CAA GTG G 3′)-s12r (5′ GKT AGT CTT RMH AGG GTC A 3′) are used for the first and A10-7R (5′ CTG RTT TGT TCA CAG TRT TG 3′) are used for the second PCR; for the middle fragment primers 6 F (5′ CCG CGG TAA TAC CAG CTC 3′)-s17r are used for the first and 6F-15A (5′ CTA AGA ACG GCC ATG CAC CAC C 3′) are used for the second amplification. The 3′ end fragment was amplified by using the barcoding primers mentioned above^38^. Thirty-five and 25 cycles were performed for the first and the second PCR, with an annealing temperature of 50 °C and 52 °C, respectively. The amplified PCR products were purified using the High Pure PCR Cleanup Micro Kit (Roche Diagnostics). Most amplified PCR products were cloned prior to sequencing using the TOPO TA Cloning Kit (Invitrogen) following the manufacturer’s instructions and transformed into competent *E. coli*. Sequencing reactions were performed using the BigDye Terminator v3.1 Cycle Sequencing Kit (Applied Biosystems) and analyzed on a 3130XL Genetic Analyzer (Applied Biosystems). All sequences present in our dataset were obtained by Sanger sequencing including those generated by other researchers.

### Data acquisition from NCBI

**Fig. 3:**
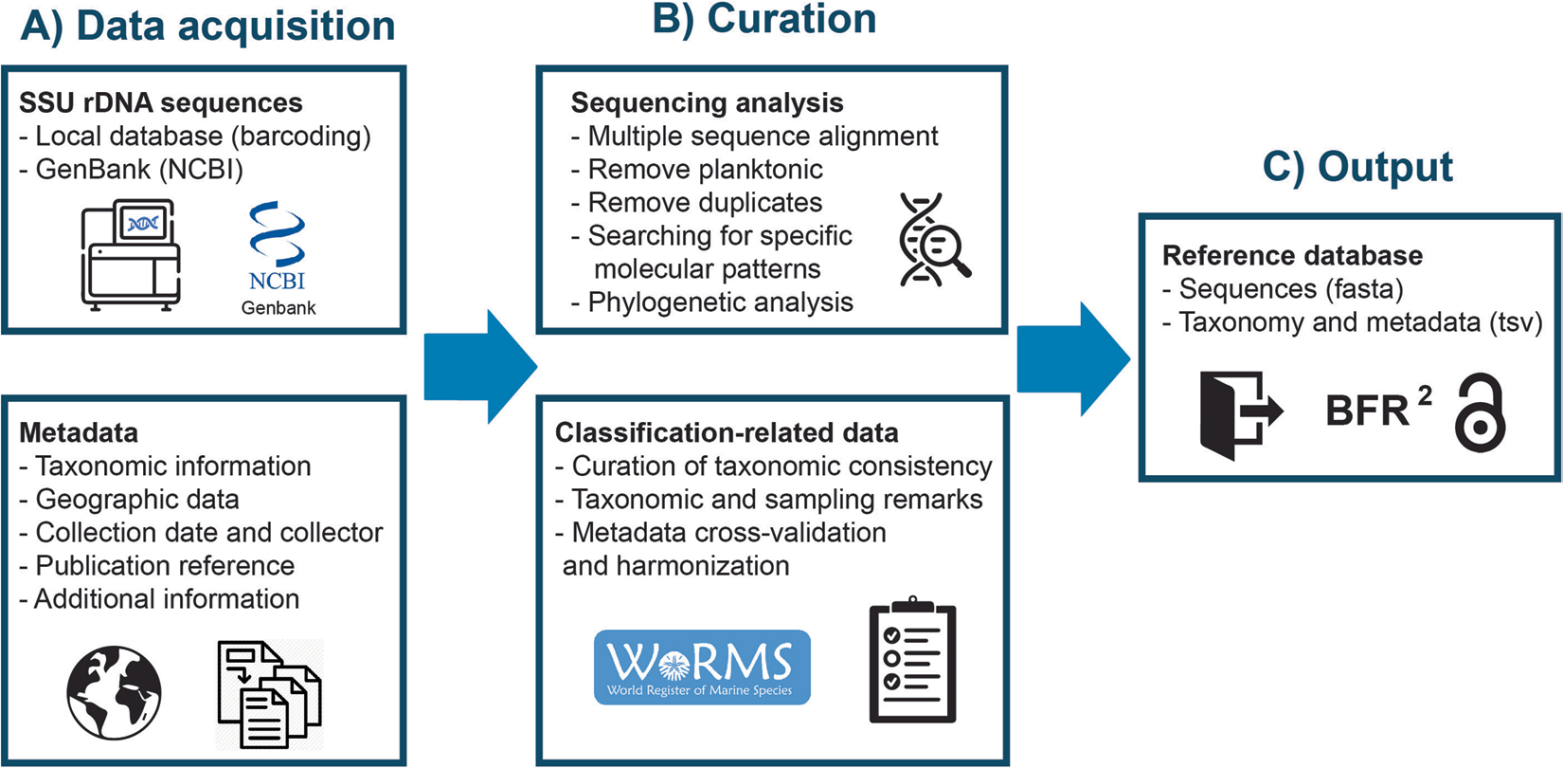
Workflow of data acquisition (**A**) and curation (**B**), including the operations carried out at each step, and the reference dataset as output (**C**).

The dataset of sequences from foraminiferal DNA collection at the University of Geneva was completed by adding 867 sequences from the nucleotide dataset of the National Center for Biotechnology Information (NCBI) until April 2024. We implemented strict criteria for NCBI sequence selection and curation procedures of the BFR2 dataset (Fig. 3). The initial criteria were as follows: (1) sequences were obtained from isolated specimens and environmental sequences were excluded; (2) sequences covered hypervariable regions 37 F and 41 F (Fig. 2B), which was based on the “GACAG” motif delimitating the foraminifera-specific 37 F region^20^.

### Data curation and phylogenetic reconstruction

Data curation started with a check for the presence of planktonic foraminifera based on the updated PFR^2^ dataset^35,36^. All sequences identified as planktonic foraminifera were removed.

The next step of data curation included checking the quality and length of sequences and insertion into an alignment. A constrained phylogenetic analysis was then used to check that sequences belonging to the same class, order, etc. were assigned to the same taxonomic levels. The alignment and phylogenetic tree were affected by inclusion of complete and partial sequences, as well as multiple identical clones. To reduce computational overhead and improve user readability, redundant sequences were removed using CD-HIT^40^, and complete (or long) sequences were trimmed according to primer set s14F1/sB (˜1500 bp, respectively). The sequences within each clade were aligned using MAFFT v.7^41^, and phylogenetic trees were inferred using RAxML^42^, based on the nucleotide substitution model that best fit the alignment data. If sequences were branching in an incongruent taxonomic clade, their identification was manually checked and curated by a combination of morphological and genetic features. If discrepancies could not be resolved, problematic sequences were removed and then the remaining sequences were re-analyzed, and trees updated. The sequences that did not match the original morphospecies they were obtained from were considered as originating from other foraminiferal species present in the isolate. These sequences were labeled as “squatters”. For the isolates, for which partial and complete sequences have been submitted separately resulting in different accession numbers, both numbers have been included.

The final step of data curation consisted of harmonizing taxonomic data and metadata of sequencing sets to create a better reference dataset for further barcoding/metabarcoding studies. The final version includes a curated reference dataset with internal and GenBank accession numbers, curated taxonomic string, and curated metadata. All contextual data are provided in a tab-delimited file^43^.

## Data Records

The BFR^2^ dataset is freely available to use for DNA barcoding or metabarcoding surveys, is permanently stored, and is made available via the FAIR open platform Zenodo^43^. The current BFR^2^ release consists of two files: (1) a tsv table containing the taxonomic and other information about the 18S rDNA sequences included in the release; and (2) a fasta file containing the full sequences.

The dataset consists of three main parts: basic sequence information, curated taxonomy, and sequence metadata. Each of these parts is subdivided. Basic sequence information comprises a unique BFR2 number for each sequence as well as the corresponding sequence and its length. Curated taxonomy follows the classification proposed in WoRMS^1^ and assembles sequences according to the three main classes Monothalamea, Tubothalamea, and Globothalamea. Each sequence is further assigned to an order, suborder, or clade, family, genus, and species. Sequence information contains also isolate numbers that are unique for each DNA extract and clone numbers for sequences derived from cloned PCR products. All sequences have been deposited at NCBI. No genomic data were generated for this manuscript. The metadata information includes coordinates, year of collection, and the name of the person who collected the foraminiferal specimens. Sampling sites specify the biogeographic region where specimens have been collected. References have been added for published sequences, consisting of the title, journal, and first author of the according publication. Sequences submitted to NCBI that are unpublished are indicated by the first name of the submission author. Taxonomic remarks include information about sequences identified as squatters and sequences obtained from non-marine foraminifera. Sampling remarks provide additional information about sampling cruises or expeditions, if available. We plan to update the dataset at a regular basis once per year.

## Technical Validation

The dataset construction was based upon a local dataset obtained from extracted foraminiferal specimens (isolates) stored in the collection of the Department of Genetics and Evolution at the University of Geneva (MH and JP) and sequences downloaded from NCBI (https://www.ncbi.nlm.nih.gov). Each entry was manually checked to correspond to the inclusion criteria before applying the curation process described above. Each sequence was identified by a unique BFR^2^ number. The sequences downloaded from NCBI contained their accession number allowing the end user to verify their original source.

## Data Availability

No custom code was used.
